# Malignant transformation of phyllodes tumor: a case report and review of literature

**DOI:** 10.1002/ccr3.1428

**Published:** 2018-02-25

**Authors:** Suragit Pornchai, Prakasit Chirappapha, Wiriya Pipatsakulroj, Panuwat Lertsithichai, Watoo Vassanasiri, Chomporn Sitathanee, Youwanush Kongdan, Thongchai Sukarayothin, Monchai Leesombatpaiboon

**Affiliations:** ^1^ Department of Surgery Faculty of Medicine Ramathibodi Hospital Mahidol University Bangkok Thailand; ^2^ Department of Pathology Faculty of Medicine, Ramathibodi Hospital Mahidol University Bangkok Thailand; ^3^ Department of Radiology Faculty of Medicine Ramathibodi Hospital Mahidol University Bangkok Thailand

**Keywords:** Fibroepithelial lesions, fibroepithelial tumors, giant fibroadenoma, juvenile fibroadenoma, malignant transformation, phyllodes tumor, recurrent tumors

## Abstract

Malignant phyllodes may transform from benign phyllodes; low‐aggressive malignant phyllodes tumor is manageable by locally wide excision.

## Introduction

The transformation of a benign phyllodes tumor into a malignant phyllodes tumor is very uncommon and unpredictable. We report a case of a 54‐year‐old woman with postlocal excision proven benign phyllodes tumor that transformed into a malignant phyllodes tumor after 36 months of surgery. We present the ultrasound and mammography images and the pathology slides of the surgical wide excision specimen. It has been suggested that mutation of residual tumor cells caused the malignant transformation. Complete surgical excision with adequate margin is essential to decrease the risk of recurrence and the malignant transformation of phyllodes tumors.

Phyllodes tumors are rare fibroepithelial breast tumors and are found in approximately 1% of primary breast tumors 1, 2. Phyllodes tumors are initially described as “cystosarcoma phyllodes” by Johannes Müller in 1838 3, and these tumors have as many as 62 different synonyms 3. Phyllodes tumor's structural pathology is similar to fibroadenoma; however, phyllodes tumors have an increased stromal hypercellularity with typical leaflike projection from histopathology 2.

The size of the tumor that has been reported ranged from 1 to 40 cm 4. Approximately 20% of patients had a mass larger than 10 cm 5. Triple assessment, that is, clinical, radiological, and histological examination, forms the fundamental basic evaluation for such lesions. The diagnosis of phyllodes tumor is based on the criteria defined by the World Health Organization in 2003, and this includes the degree of stromal hypercellularity, mitoses, cytologic atypia, stromal overgrowth, and the nature of the tumor border. The WHO classification distinguished phyllodes tumor into three histological subtypes: benign, borderline, and malignant 2, which account for 58.4–74.6%, 15.0–16.1%, and 9.3–31% of all phyllodes tumors, respectively 6, 7, 8.The behavior is unpredictable, and the distinction between benign, borderline, and malignant tumors is often difficult and does not always reflect the clinical behavior 9. The mainstay treatment of phyllodes tumor is surgery, with an adequate margin of at least 1 cm 10. In some cases where the mass was large, immediate reconstruction was required to improve the cosmetic outcome 10, 11.

The prognosis of phyllodes tumor is quite good. In a retrospective study of 101 patients treated between 1944 and 1998, the overall survival rate of patients with benign and borderline tumors was 91% at 5 years. The five‐year survival rate for malignant phyllodes tumors was 82% 12.

To date, the local recurrence rate in phyllodes tumor is approximately 15% 13, 14, 15. Local recurrence usually occurs within the first few years following surgery, especially if it was with incomplete excision 7. Tumors typically recur locally within 2 years of initial excision 16. Approximately 15% have a propensity to recur locally and about 10% chance of distant recurrence 16.

Malignant transformation of phyllodes tumor is a very rare form of breast cancer. Only some reports show malignant transformation from a benign phyllodes tumor 13, 17, 18, 19, 20. We report one case of a benign phyllodes tumor that transformed into a malignant phyllodes tumor during its recurrence.

## Case Report

**Figure 1:**
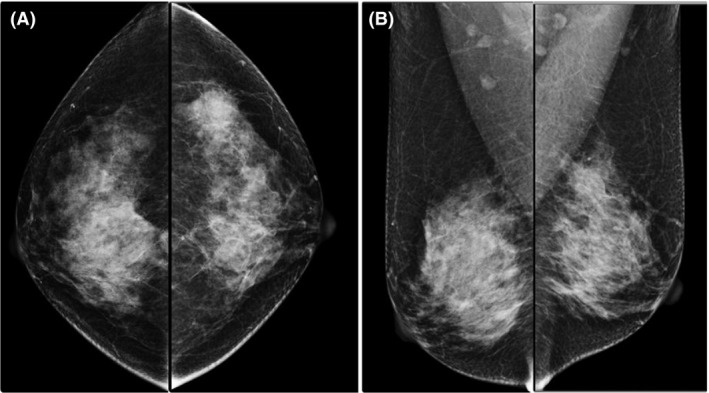
Mammogram in October 2015 (A, CC view; B, MLO view), demonstrating a mass shadow in left upper outer quadrant without calcification.

**Figure 2:**
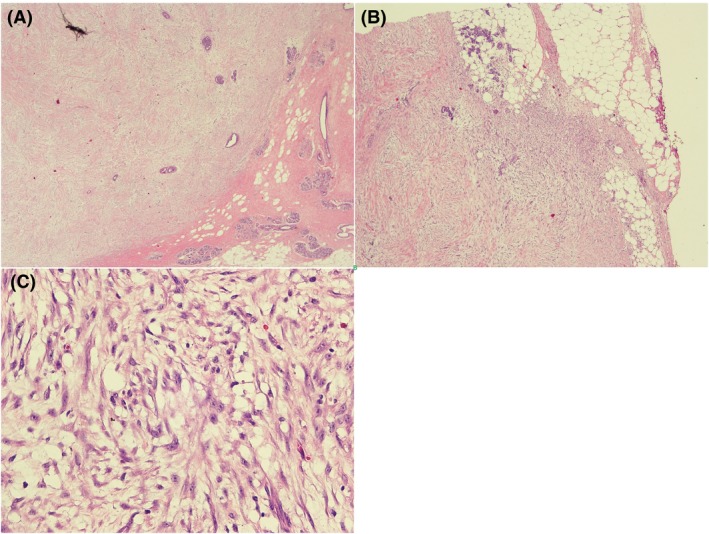
Histology slide from October 2015 showed. (A) Relatively circumscribed fibroepithelial lesion with stromal expansion (H&E, 4). (B) Infiltration into the surrounding adipocytes (H&E, 10). (C) The stroma shows moderate hypercellularity and moderate atypia (H&E, 400).

A 54‐year‐old female presented to a private hospital in Bangkok, Thailand, in October 2015 with a 3‐cm lump in her left breast. Her mammogram in October 2015 showed a mass at the upper outer quadrant of the left breast without calcification (Fig. 1). A focused ultrasound revealed a shaggy border mass size 2.5 cm. Core needle biopsy was performed, and the histopathological report was spindle cell lesion. The mass was excised in October 2015. Gross examination revealed an ill‐defined firm gray‐white mass, measuring 2.8 × 2.4 × 2.2 cm, close to resection margin. Microscopic examination showed a relatively circumscribed fibroepithelial lesion with stromal expansion. Multifoci of infiltration into the surrounding adipocytes and entrapment of normal mammary lobule were also noted. The stroma showed moderate hypercellularity and moderate nuclear atypia. No stromal overgrowth was detected. The margin displayed small tumor buds protruding into the surrounding tissue. Mitotic count is 2/10 high power field (HPF) (Fig. 2). The final pathological reported was benign phyllodes tumor. The result of molecular study was CD34 negative, CD117 positive, p53 weak‐to‐moderate staining in approximately 50%, Ki67 = 3%, EGFR moderate membranous straining in approximately 60%, Beta‐catenin no nuclear staining, Bcl2 positive in few cell (<1%), and ER negative.

**Figure 3:**
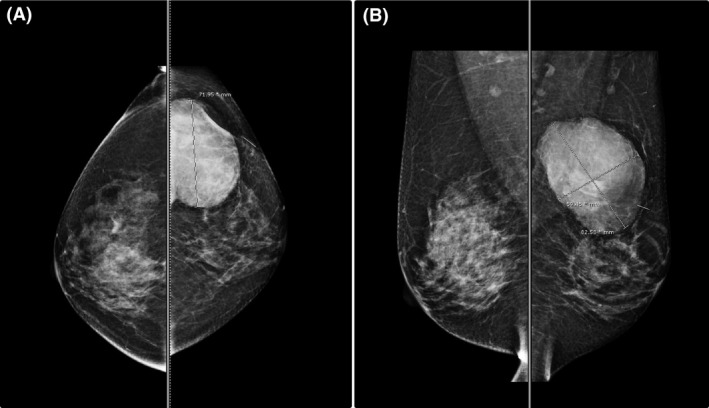
Mammogram in April 2017 (A, CC view; B, MLO view), demonstrating a large lobulated mass at upper outer quadrant of the left breast, 8.3 × 5.9 × 7.2 cm in size corresponding to the palpable mass.

**Figure 4:**
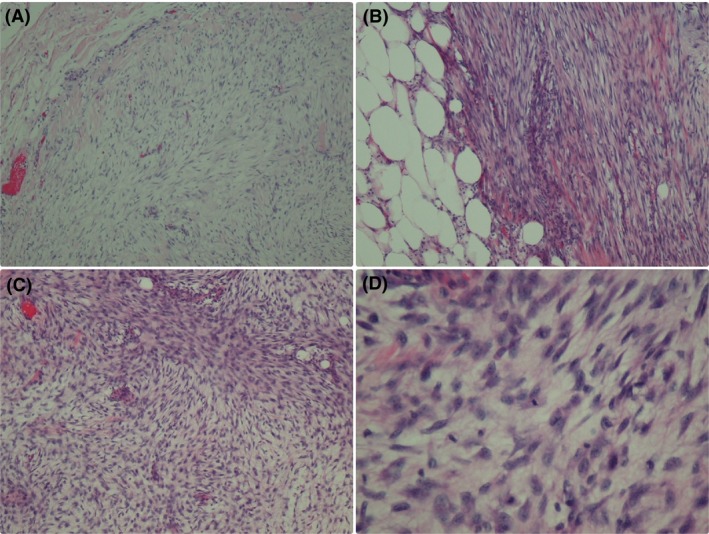
Histology slide from April 2017 showed. (A) Stromal overgrowth (H&E, 4). (B) Infiltration into the surrounding adipocytes (H&E, 10). (C) Moderate‐to‐marked stromal hypercellularity, moderate‐to‐marked stromal atypia, and increased mitotic figures (H&E, 40). (D) Moderate‐to‐marked stromal hypercellularity, moderate‐to‐marked stromal atypia, and increased mitotic figures (H&E, 400).

In April 2017, she revisited the hospital due to a rapidly growing mass at the site of prior excision for 3 month. On physical examination, the patient was found to have a 7‐cm firm mobile mass in the 2 o'clock position of the left breast without evidence of any lymphadenopathy. Mammogram in April 2017 showed a large lobulated mass at the upper outer quadrant of the left breast (Fig. 3). A focused breast ultrasound also showed a large lobulated solid‐cystic mass at the surgical bed (8.3 × 5.9 × 7.2 cm in size). We review the previous histopathology specimen from 2015, and our pathologist suggested that it could have been borderline instead of benign phyllodes tumor. The patient underwent wide local excision with wedging of pectoralis major muscle. Pathology revealed phyllodes tumor 7.5 cm in maximum diameter, invading the pectoralis major muscle with free all margins at least 1 cm. The tumor showed moderate‐to‐marked stromal hypercellularity, moderate‐to‐marked stromal atypia, focal ill‐defined border, prominent stromal overgrowth, and mitotic count 5/10 HPF (Fig. 4). The final pathological reported this time is malignant phyllodes tumor. The result of molecular study was CD34 negative, CD117 negative, p53 moderate‐to‐strong staining in approximately 80%, Ki67 = 7%, EGFR moderate membranous straining in approximately 60%, Beta‐catenin no nuclear staining, Bcl2 negative, and ER negative.

## Discussion

Local excision with close margin is the treatment for most women diagnosed with benign phyllodes in order to preserve the breast parenchyma. Therefore, some residual tumor cells may still remain. It had been suggested that mutation of residual benign phyllodes tumor cell caused malignant transformation 21. Generally, recurrent tumors are histologically similar to the primary tumors, but they are often more cellular, with focal area of atypical cell 21. It is important that clinicians are aware that not only benign phyllodes tumor may recur but the recurrences will also occasionally show sufficient atypia to warrant the diagnosis of malignant phyllodes tumor 21. In our case, the tumor recurred with increased degree of stromal atypia, stromal cellularity, mitotic count, and prominent stromal overgrowth.

Fibroepithelial tumors of breast, including fibroadenoma and phyllodes tumor, are biphasic neoplasms characterized by proliferation of both epithelial and stromal components. Phyllodes tumor of various grades shares some overlapping morphology, but they are significantly different in clinicopathologic features and clinical behavior 22 (Table 1). In our case, the clinicopathologic features of the primary tumor include moderate stromal hypercellularity, moderate stromal atypia, focally ill‐defined border, no stromal overgrowth, and a mitotic count of 2/10 HPF. These findings are compatible with those of benign‐to‐borderline phyllodes tumor. The clinicopathologic features of the recurrent tumor include moderate‐to‐marked stromal hypercellularity, moderate‐to‐marked stromal atypia, focally ill‐defined border, prominent stromal overgrowth, and a mitotic count of 5/10 HPF which is mostly compatible with the diagnosis of malignant phyllodes.

**Table 1:** Clinicopathologic features of benign, borderline, and malignant phyllodes tumor

Clinicopathologic features	Benign	Borderline	Malignant
Mitoses/10HPF	1–3	4–9	≥10
Cellularity	Modest	Modest	Mark
Pleomorphism	Little	Moderate	Mark
Border	Pushing	Intermediate	Infiltrating
Stroma	Uniform	Heterogeneous	Overgrowth
Ki67 index	Low	Intermediate	High

Some studies had described the mechanism of malignant transformation 23, 24. A clonal analysis of phyllodes tumors showed that it consists of polyclonal epithelial cells and monoclonal stromal cells. The stromal cellularity of phyllodes tumor with high malignant potential increases, and its epithelial cellularity decreases as the size of tumor increases. Therefore, phyllodes tumor can be regarded as stromal cells neoplasm. A single stromal cell can undergo mutation and develops into a malignant phyllodes tumor composed mainly of monoclonal stomal cells and partially of polyclonal epithelial cells 23, 24. In our case, the tumor was composed of both epithelial and stromal cells, but mainly of stromal cells.

Molecular profile of the tumor may help in evaluating malignant transformation. In benign phyllodes tumors, the presence of mitotic figures within the periductal stroma was opposed to stroma away from the epithelium. It is believed that the growth of tumor comes from the epithelial–stromal interaction process including Wnt‐APC‐Beta‐catenin pathway and Estrogen receptor pathway 25. Molecular studies have shown that the increase in the expression of Beta‐catenin or estrogen receptor is associated with benign phyllodes tumors, but if there is a decrease in the expression, it is associated with malignant phyllodes tumors 26, 27. In our study, both Beta‐catenin and estrogen receptor were negative for both the primary and the recurrence tumors. In our study, bcl‐2 is an inner mitochondrial membrane protein that inhibits apoptosis 28. It was first recognized in follicular lymphoma in which a 14;18 translocation results in overexpression of bcl‐2 29, and expression was subsequently shown in other types of lymphoma and in some normal lymphocytes 30. bcl‐2 expression is associated with epithelium that undergoes hyperplasia and atrophy usually under hormonal control including breast and prostate. In the normal breast and benign phyllodes tumor, there were strong staining of lymphocytes and moderate‐to‐strong staining of normal epithelium 30. In the one studied: 26 phyllodes tumors (15 benign, 4 borderline, 9 and 7 malignant), there was less bcl‐2 expression in malignant (median 1.5%) than in benign and borderline phyllodes tumors 31. In our study, bcl‐2 was positive in few cell (<1%) in primary tumor and negative in recurrence tumor.

In malignant phyllodes tumor, the growth depends on the stromal component, which is molecular profile, and gene mutation like other types of cancer includes p53, Ki67, CD117 (c‐kit), EGFR, and CD34.

### P53

The p53 is a product of a tumor suppressor gene, located on chromosome 17p13.1 32. Its mutations are one of the most common genetic abnormalities in cancer, for which it is deemed a useful prognostic predictor, and its mutant forms are reflected by increased p53 staining on immunohistochemistry 32. Stromal p53 expression has almost consistently been reported to increase significantly with malignant phyllodes tumor 33, which is represented mainly by stromal hypercellularity and overgrowth 32. No study reported any correlation with recurrent disease 32, 33. The significant increase in p53 expression would mostly be between benign phyllodes tumors and borderline‐to‐malignant tumors, with no significant difference in the latter two categories 32, 34. In our study, p53 was weak‐to‐moderate staining in approximately 50% in primary tumor and moderate‐to‐strong staining in approximately 80% in recurrence tumor.

### Ki‐67

The nuclear protein Ki‐67 is a cell proliferation marker expressed only in cycling cells. As a result, quantitative assessment of Ki‐67 nuclear staining on tumor samples provides a good estimate of the proliferation index of individual tumors. Ki‐67 index increased in borderline and especially in malignant PTs 35. Ki‐67‐labeling indices have ranged from 1.3% to 4.7%, 6% to 26%, and 12% to 50%, for benign, borderline, and malignant phyllodes tumors, respectively 36. Ki‐67 proliferation index has been significantly correlated with disease‐free and overall survival rates 37. In our study, Ki67 was 3% in primary tumor and 7% in recurrence tumor.

### CD117 (c‐kit)

CD117 is a transmembrane protein of the type III receptor tyrosine kinase family. It is crucial in regulating cell survival, proliferation, and differentiation. It is recognized as a protooncogene with frequent activating mutations and/or overexpression in several types of neoplasms, including gastrointestinal stromal tumors, seminomas, melanomas, and hematopoietic malignancies. CD117 has thus been considered another possible contributor to stromal proliferation in the phyllodes tumors 32, 33, 38, 39 presumably participating in the process of cell cycle progression, and synergistic with p53 protein, with which its immunohistostaining has also been significantly correlated 32, 38, 40. In other studies found, there is no association between CD117 expression and tumor grade, and furthermore, KIT‐activating mutations have not been found in PTs 41, 42, 43. In our study, CD117 was positive in primary tumor and negative in recurrence tumor.

### CD34

CD34 is a type I transmembrane glycoprotein expressed on hemopoietic stem and progenitor cells, endothelial cells, and a subset of fibroblast and bone marrow progenitor cells, and is expressed in many mesenchymal tumors. CD34 coexists with c‐kit in GIST and its possible coexistence with c‐kit in phyllodes tumors 40. In one study, it has shown that CD34 expression in 8 of 12 malignant tumors and only one in benign tumor 40. Just like p53, Ki‐67, and c‐kit, the ability of CD34 to predict outcome, however, was also described as questionable 40. In our study, CD34 was negative in both primary tumor and recurrence tumor.

### EGFR

Epidermal growth factor receptor (EGFR) is said to mediate tumor formation and progression pathways intracellularly, via ras‐activated mitogen‐activated protein kinase, phosphatidylinositol 3‐kinase/AKT, and phospholipase C pathways that modulate cell motility, adhesion, and proliferation 44. EGFR was further associated with stromal cellularity and overgrowth, nuclear pleomorphism, mitosis, infiltrative margins, and size 45. Immunopositivity for EGFR in the stromal cells was detected in 19% of 58 phyllodes tumors (75% of all malignant tumors) 46. In our study, EGFR was moderate membranous straining in approximately 60% in both primary tumor and recurrence tumor. Conclusion of molecular profile of malignant transformation of phyllodes tumor is shown in Table 2.

**Table 2:** Correlations of molecular profile changes in the progression of malignant phyllodes tumors

Increase expression	Decrease expression
p53	Bcl2
CD34	Estrogen receptor
EGFR	Beta‐catenin
c‐kit	
Ki67	

The treatment of phyllodes tumor is local excision to obtain adequate margin. According to most studies, the margin at least 1 cm is considered adequate. The most evidence comes from retrospective studies 5, 12, 14, 47. If inadequate margin after resection, about 20% of the phyllodes tumor will be local recurrence and may be lead to malignant transformation 48. According to the NCCN guideline, a margin 1 cm or more is considered adequate and does not relate to the histology subtype of phyllodes 49. In our study, the primary tumor was close resection margin after excised in October 2015. It may be the cause of local recurrence and malignant transformation.

Phyllodes tumors, that are local recurrence after excision. The treatment is re‐excision if it is possible to achieve an adequate margin of 1 cm and acceptable cosmetic outcome. However, if the surgery affects the cosmetic outcome, mastectomy should be considered 49. In some cases, that mastectomy cannot cover the defect. The another option may be considered including skin graft, local flap, pedicle flap, or free flap to close the defect 10. In our study, the recurrence tumor was cleared free all margins at least 1 cm after wide local excision with wedging of pectoralis major muscle in April 2017**.** In October 2017, this patient is not recurrence.

## Conclusion

Based on the data from previous literature and the case presented in this paper, it can be concluded that benign or borderline phyllodes tumor can progress to malignant phyllodes tumor after local excision, as mutation of residual tumor cell is the source of malignant transformation. The most widely accepted theory is p53 mutation. Complete surgical excision with adequate margin is essential to decrease the risk of recurrence and malignant transformation of phyllodes tumors.

## Conflict of Interest

None declared.

## Authorship

SP: collected data, writing of the manuscript, literature review, submitted the manuscript for publication. PC: reviewed and recommended edits of the manuscript, supervised the whole study process, and approved the final manuscript. WP: revised pathologic images, data and interpretation molecular profile, supervised the whole study process, and approved the final manuscript. WV: revised the English version of the manuscript. PL, CS, YK, TS, and ML: supervised the whole study process and approved the final manuscript.

## References

[1] Kraemer, B. , J. Hoffmann , C. Roehm , C. Gall , D. Wallwiener , and U. Krainick‐Strobel . 2007 Cystosarcoma phyllodes of the breast: a rare diagnosis: case studies and review of literature. Arch. Gynecol. Obstet. 276:649–653. 17549503 10.1007/s00404-007-0393-6

[2] Lakhani, S. R. 2012 World Health Organization classification of tumours, 4th ed. World Health Organization‐IARC, Lyon.

[3] Fiks, A. 1981 Cystosarcoma phyllodes of the mammary gland–Muller's tumor. For the 180th birthday of Johannes Muller. Virchows Archiv. A 392:1–6. 10.1007/BF00430543 6269275

[4] Hawkins, R. E. , J. B. Schofield , C. Fisher , E. Wiltshaw , and J. A. McKinna . 1992 The clinical and histologic criteria that predict metastases from cystosarcoma phyllodes. Cancer 69:141–147. 1309302 10.1002/1097-0142(19920101)69:1<141::aid-cncr2820690125>3.0.co;2-1

[5] Reinfuss, M. , J. Mitus , K. Duda , A. Stelmach , J. Rys , and K. Smolak . 1996 The treatment and prognosis of patients with phyllodes tumor of the breast: an analysis of 170 cases. Cancer 77:910–916. 8608483 10.1002/(sici)1097-0142(19960301)77:5<910::aid-cncr16>3.0.co;2-6

[6] Tan, P. H. , T. Jayabaskar , K. L. Chuah , H. Y. Lee , Y. Tan , M. Hilmy , et al. 2005 Phyllodes tumors of the breast: the role of pathologic parameters. Am. J. Clin. Pathol. 123:529–540. 15743740 10.1309/U6DV-BFM8-1MLJ-C1FN

[7] Karim, R. Z. , S. K. Gerega , Y. H. Yang , A. Spillane , H. Carmalt , R. A. Scolyer , et al. 2009 Phyllodes tumours of the breast: a clinicopathological analysis of 65 cases from a single institution. Breast 18:165–170. 19329316 10.1016/j.breast.2009.03.001

[8] Taira, N. , D. Takabatake , K. Aogi , S. Ohsumi , S. Takashima , R. Nishimura , et al. 2007 Phyllodes tumor of the breast: stromal overgrowth and histological classification are useful prognosis‐predictive factors for local recurrence in patients with a positive surgical margin. Jpn. J. Clin. Oncol. 37:730–736. 17932112 10.1093/jjco/hym099

[9] Sotheran, W. , J. Domjan , M. Jeffrey , M. H. Wise , and P. M. Perry . 2005 Phyllodes tumours of the breast–a retrospective study from 1982–2000 of 50 cases in Portsmouth. Ann. R. Coll. Surg. Engl. 87:339–344. 16176692 10.1308/003588405X51128 PMC1963973

[10] Chirappapha, P. , P. Lertsithichai , T. Sukarayothin , M. Leesombatpaiboon , C. Supsamutchai , and Y. Kongdan . 2016 Oncoplastic techniques in breast surgery for special therapeutic problems. Gland Surg. 5:75–82. 26855912 10.3978/j.issn.2227-684X.2015.05.04 PMC4716860

[11] Fang, C. L. , C. H. Hsu , and C. W. Tu . 2017 The reconstruction choice for giant phyllodes tumor of breast: bi‐pedicled deep inferior epigastric perforator flap. Aesthetic Plast. Surg. 41:768–772. 28130560 10.1007/s00266-017-0792-4

[12] Chaney, A. W. , A. Pollack , M. D. McNeese , G. K. Zagars , P. W. Pisters , R. E. Pollock , et al. 2000 Primary treatment of cystosarcoma phyllodes of the breast. Cancer 89:1502–1511. 11013364 10.1002/1097-0142(20001001)89:7<1502::aid-cncr13>3.0.co;2-p

[13] Parker, S. J. , and S. A. Harries . 2001 Phyllodes tumours. Postgrad. Med. J. 77:428–435. 11423590 10.1136/pmj.77.909.428 PMC1760996

[14] Barth, R. J. Jr , W. A. Wells , S. E. Mitchell , and B. F. Cole . 2009 A prospective, multi‐institutional study of adjuvant radiotherapy after resection of malignant phyllodes tumors. Ann. Surg. Oncol. 16:2288–2294. 19424757 10.1245/s10434-009-0489-2 PMC5053421

[15] Tan, P. H. , A. A. Thike , W. J. Tan , M. M. Thu , I. Busmanis , H. Li , et al. 2012 Predicting clinical behaviour of breast phyllodes tumours: a nomogram based on histological criteria and surgical margins. J. Clin. Pathol. 65:69–76. 22049216 10.1136/jclinpath-2011-200368

[16] Telli, M. L. , K. C. Horst , A. E. Guardino , F. M. Dirbas , and R. W. Carlson . 2007 Phyllodes tumors of the breast: natural history, diagnosis, and treatment. J. Natl. Comprehens. Cancer Netw. 5:324–330. 10.6004/jnccn.2007.0027 17439760

[17] West, T. L. , L. H. Weiland , and O. T. Clagett . 1971 Cystosarcoma phyllodes. Ann. Surg. 173:520–528. 4324792 10.1097/00000658-197104000-00007 PMC1397400

[18] Blichert‐Toft, M. , J. P. Hansen , O. H. Hansen , and T. Schiodt . 1975 Clinical course of cystosarcoma phyllodes related to histologic appearance. Surg. Gynecol. Obstet. 140:929–932. 165582

[19] Liu, T. P. J. K. , and T. L. Yang . 1995 Surgical treatment of phyllodes tumors of the breast: retrospective review of 66 cases. J. Chin. Oncol. Soc. 11:35–42. 10.1002/jso.20334 16118768

[20] Lin, C. C. , H. W. Chang , C. Y. Lin , C. F. Chiu , and S. P. Yeh . 2013 The clinical features and prognosis of phyllodes tumors: a single institution experience in Taiwan. Int. J. Clin. Oncol. 18:614–620. 22773245 10.1007/s10147-012-0442-4

[21] Hajdu, S. I. , M. H. Espinosa , and G. F. Robbins . 1976 Recurrent cystosarcoma phyllodes: a clinicopathologic study of 32 cases. Cancer 38:1402–1406. 182356 10.1002/1097-0142(197609)38:3<1402::aid-cncr2820380346>3.0.co;2-9

[22] Yang, X. , D. Kandil , E. F. Cosar , and A. Khan . 2014 Fibroepithelial tumors of the breast: pathologic and immunohistochemical features and molecular mechanisms. Arch. Pathol. Lab. Med. 138:25–36. 24377809 10.5858/arpa.2012-0443-RA

[23] Noguchi, S. , K. Motomura , H. Inaji , S. Imaoka , and H. Koyama . 1993 Clonal analysis of fibroadenoma and phyllodes tumor of the breast. Can. Res. 53:4071–4074. 8395336

[24] Noguchi, S. , H. Yokouchi , T. Aihara , K. Motomura , H. Inaji , S. Imaoka , et al. 1995 Progression of fibroadenoma to phyllodes tumor demonstrated by clonal analysis. Cancer 76:1779–1785. 8625047 10.1002/1097-0142(19951115)76:10<1779::aid-cncr2820761015>3.0.co;2-0

[25] Sawhney, N. , N. Garrahan , A. G. Douglas‐Jones , and E. D. Williams . 1992 Epithelial–stromal interactions in tumors. A morphologic study of fibroepithelial tumors of the breast. Cancer 70:2115–2120. 1327488 10.1002/1097-0142(19921015)70:8<2115::aid-cncr2820700818>3.0.co;2-k

[26] Sawyer, E. J. , A. M. Hanby , A. J. Rowan , C. E. Gillett , R. E. Thomas , R. Poulsom , et al. 2002 The Wnt pathway, epithelial‐stromal interactions, and malignant progression in phyllodes tumours. J. Pathol. 196:437–444. 11920740 10.1002/path.1067

[27] Tse, G. M. , C. S. Lee , F. Y. Kung , R. A. Scolyer , B. K. Law , T. S. Lau , et al. 2002 Hormonal receptors expression in epithelial cells of mammary phyllodes tumors correlates with pathologic grade of the tumor: a multicenter study of 143 cases. Am. J. Clin. Pathol. 118:522–526. 12375638 10.1309/D206-DLF8-WDNC-XJ8K

[28] Hockenbery, D. , G. Nunez , C. Milliman , R. D. Schreiber , and S. J. Korsmeyer . 1990 Bcl‐2 is an inner mitochondrial membrane protein that blocks programmed cell death. Nature 348:334–336. 2250705 10.1038/348334a0

[29] Tsujimoto, Y. , L. R. Finger , J. Yunis , P. C. Nowell , and C. M. Croce . 1984 Cloning of the chromosome breakpoint of neoplastic B cells with the t(14;18) chromosome translocation. Science 226:1097–1099. 6093263 10.1126/science.6093263

[30] Pezzella, F. , A. G. Tse , J. L. Cordell , K. A. Pulford , K. C. Gatter , and D. Y. Mason . 1990 Expression of the bcl‐2 oncogene protein is not specific for the 14;18 chromosomal translocation. Am. J. Pathol. 137:225–232. 2201196 PMC1877598

[31] Moore, T. , and A. H. Lee . 2001 Expression of CD34 and bcl‐2 in phyllodes tumours, fibroadenomas and spindle cell lesions of the breast. Histopathology 38:62–67. 11135048 10.1046/j.1365-2559.2001.01053.x

[32] Tan, P. H. , T. Jayabaskar , G. Yip , Y. Tan , M. Hilmy , S. Selvarajan , et al. 2005 p53 and c‐kit (CD117) protein expression as prognostic indicators in breast phyllodes tumors: a tissue microarray study. Mod. Pathol. 18:1527–1534. 16258510 10.1038/modpathol.3800488

[33] Tsea, G. M. K. , and P. H. Tanb . 2005 Recent advances in the pathology of fibroepithelial tumors of the breast. Curr. Diagnost. Pathol. 11:426–434.

[34] Millar, E. K. , J. Beretov , P. Marr , M. Sarris , R. A. Clarke , J. H. Kearsley , et al. 1999 Malignant phyllodes tumours of the breast display increased stromal p53 protein expression. Histopathology 34:491–496. 10383692 10.1111/j.1365-2559.1999.00666.x

[35] Yang, X. , B. Ustun , S. Goodman , D. Kandil , and A. Khan . 2012 Differentiating borderline and malignant phyllodes tumor of the breast. Modern Pathol. 25:75A.

[36] Esposito, N. N. , D. Mohan , A. Brufsky , Y. Lin , M. Kapali , and D. J. Dabbs . 2006 Phyllodes tumor: a clinicopathologic and immunohistochemical study of 30 cases. Arch. Pathol. Lab. Med. 130:1516–1521. 17090194 10.5858/2006-130-1516-PTACAI

[37] Niezabitowski, A. , B. Lackowska , J. Rys , A. Kruczak , T. Kowalska , J. Mitus , et al. 2001 Prognostic evaluation of proliferative activity and DNA content in the phyllodes tumor of the breast: immunohistochemical and flow cytometric study of 118 cases. Breast Cancer Res. Treat. 65:77–85. 11245343 10.1023/a:1006457304526

[38] Tse, G. M. , T. C. Putti , P. C. Lui , A. W. Lo , R. A. Scolyer , B. K. Law , et al. 2004 Increased c‐kit (CD117) expression in malignant mammary phyllodes tumors. Mod. Pathol. 17:827–831. 15044924 10.1038/modpathol.3800125

[39] Carvalho, S. , A. O. Silva , F. Milanezi , S. Ricardo , D. Leitao , I. Amendoeira , et al. 2004 c‐KIT and PDGFRA in breast phyllodes tumours: overexpression without mutations? J. Clin. Pathol. 57:1075–1079. 15452163 10.1136/jcp.2004.016378 PMC1770449

[40] Chen, C. M. , C. J. Chen , C. L. Chang , J. S. Shyu , H. F. Hsieh , and H. J. Harn . 2000 CD34, CD117, and actin expression in phyllodes tumor of the breast. J. Surg. Res. 94:84–91. 11104647 10.1006/jsre.2000.6001

[41] Korcheva, V. B. , J. Levine , C. Beadling , A. Warrick , G. Countryman , N. R. Olson , et al. 2011 Immunohistochemical and molecular markers in breast phyllodes tumors. Appl. Immunohistochem. Mol. Morphol. 19:119–125. 21030860 10.1097/PAI.0b013e3181f5349a

[42] Djordjevic, B. , and W. M. Hanna . 2008 Expression of c‐kit in fibroepithelial lesions of the breast is a mast cell phenomenon. Mod. Pathol. 21:1238–1245. 18500266 10.1038/modpathol.2008.78

[43] Bose, P. , S. T. Dunn , J. Yang , R. Allen , C. El‐Khoury , and A. Tfayli . 2010 c‐Kit expression and mutations in phyllodes tumors of the breast. Anticancer Res. 30:4731–4736. 21115932

[44] Oda, K. , Y. Matsuoka , A. Funahashi , and H. Kitano . 2005 A comprehensive pathway map of epidermal growth factor receptor signaling. Mol. Syst. Biol. 1:2005.0010. 10.1038/msb4100014 PMC1681468 16729045

[45] Tse, G. M. , P. C. Lui , J. S. Vong , K. M. Lau , T. C. Putti , R. Karim , et al. 2009 Increased epidermal growth factor receptor (EGFR) expression in malignant mammary phyllodes tumors. Breast Cancer Res. Treat. 114:441–448. 18443904 10.1007/s10549-008-0030-5

[46] Kersting, C. , A. Kuijper , H. Schmidt , J. Packeisen , C. Liedtke , N. Tidow , et al. 2006 Amplifications of the epidermal growth factor receptor gene (egfr) are common in phyllodes tumors of the breast and are associated with tumor progression. Lab. Investig. 86:54–61. 16258523 10.1038/labinvest.3700358

[47] Kapiris, I. , N. Nasiri , R. A'Hern , V. Healy , and G. P. Gui . 2001 Outcome and predictive factors of local recurrence and distant metastases following primary surgical treatment of high‐grade malignant phyllodes tumours of the breast. Eur. J. Surg. Oncol. 27:723–730. 11735168 10.1053/ejso.2001.1207

[48] Guillot, E. , B. Couturaud , F. Reyal , A. Curnier , J. Ravinet , M. Lae , et al. 2011 Management of phyllodes breast tumors. Breast J. 17:129–137. 21251125 10.1111/j.1524-4741.2010.01045.x

[49] Network NCCN Breast cancer (version 2.2017).

